# Young female sex workers lived experiences of selling sex and accessing sexual and reproductive health services in Tanzania: implications for HIV prevention

**DOI:** 10.3389/fpubh.2026.1843995

**Published:** 2026-06-10

**Authors:** Joyce Wamoyi, Kaymarlin Govender, Samuel Mgunga

**Affiliations:** 1 Department of Sexual and Reproductive Health, National Institute for Medical Research, Mwanza, Tanzania; 2 Health Economics HIV and AIDS Division (HEARD), Faculty of Law and Business Management, University of KwaZulu-Natal, Durban, South Africa

**Keywords:** HIV/AIDS, sexual and reproductive health services, sexual health, Tanzania, young female sex workers

## Abstract

**Background:**

Adolescent girls and young women who sell sex are at a heightened risk of acquiring HIV compared to those who do not. Their access to Sexual and Reproductive Health (SRH) services is also limited. This study explored the context of sex work for young female sex workers (YFSWs) and how they accessed sexual and reproductive health (SRH) services, including HIV services, to gain insights for enhancing demand for and access to these services.

**Methods:**

This study utilized an ethnographic research design involving 38 in-depth interviews and photovoice with YFSWs aged 18–24 years and six SRH program implementers. Thematic analysis was conducted with the aid of NVIVO software.

**Results:**

Five themes emerged: (1) “Selling sex in a setting where it is illegal”; (2) Nature of SRH services available to YFSWs; (3) Stigma as a barrier to accessing services; (4) Preference for differentiated type of SRH services; and (5) Peer influencers in creating demand for SRH services. Participants reported that sex work was illegal in Tanzania and highly stigmatized. YFSWs operated discreetly for fear of police arrests, and stigma and discrimination. Stigma and discrimination hindered access to SRH services. Peers were important in influencing YFSWs’ decision on whether to use or not and where and how to access the SRH services.

**Conclusion:**

YFSWS operate under stressful conditions and had limited access to SRH services. Peers of YFSWs played a key role in access to and use of SRH services. There is a need for differentiated care for YFSWs and building an enabling environment to motivate YFSW to access SRHs. Given the numerous challenges faced by YFSWs in accessing SRH services, interventions targeted at this group should focus on multi-level barriers: (a) at health facility level, provide differentiated SRH care/services that are friendly and trusted; (b) at community level, address stigma and social norms that promote gender inequalities; (c) at individual level, provide SRH knowledge and livelihood options and; and finally, (d) at policy level, address policies that criminalize sex work.

## Background

While evidence points to adolescent girls and young women being at increased risk for HIV infection, young women who sell sex face an even higher risk. On average, sex workers have 30 times greater risk of acquiring HIV than women in the general population (1). A study of 16 countries in sub-Saharan Africa found an average HIV prevalence of 37% among sex workers (2). In Tanzania, HIV prevalence among sex workers is 28% which is five times higher than the national average (3). However, approximately 32.8% of sex workers do not know their HIV status (4).

Factors such as frequent unsafe sex with multiple partners, inability to negotiate consistent condom use, and experiencing violence, criminalization and marginalization place sex workers at an elevated risk of HIV and sexually transmitted infections (5–8). Population size estimates show that there are nearly 1 million sex workers in need of services (3). Moreover, less than half of female sex workers could access at least two HIV prevention services in the past 3 months in 16 of the 30 reporting countries in recent years (4). In as much as interventions have focused at reducing HIV risk among adolescent girls and young women in sub-Saharan Africa, there is dearth of evidence on young women who sell sex in the region (7). This population has special vulnerabilities (young adulthood and the exploitative nature of selling sex) compared to other young women in the general population (5). Therefore, combining their needs with the general population of young women may not achieve much for them. They also face stigma that limits their access to SRH services thus increasing their risk for HIV but also impacting their general wellbeing and development (3, 5, 6). Moreover, young women in this age range (18–24 years) who engage in sex work may not be reached with HIV prevention and treatment services as a result of not disclosing that they engage in acts of selling sex (9).

### Context of sex work in Tanzania

Although the estimated size of the sex worker population in Tanzania between 2014 and 2017 was 160,000, the third highest in East and Southern Africa (10), there have been little success in getting intervention services to this population especially, young female sex workers (YFSWs). Tanzania also criminalizes sex work (3). Condom use has been associated with reduced risk of HIV infection among sex workers (11–13), however only 6% of female sex workers in Tanzania reported dual use of modern contraceptive and condoms, therefore increasing their risk for unplanned pregnancy and HIV infection (14).

The HIV epidemic is concentrated in particular groups such as female sex workers, but little has been done to understand the vulnerabilities of young women (aged 18–24 years) who engage in sex work in Mwanza region, Tanzania. In order to address the sexual and reproductive health needs young women who sell sex, it is key to understand the context in which they practice this. This study explored the context of sex work for young female sex workers (YFSWs) and how they accessed sexual and reproductive health (SRH) services, including HIV services, to gain insights for enhancing demand for and access to these services.

ding HIV services to garner insights for enhancing demand and access to these services.

## Methods

This study utilized an ethnographic research design involving in-depth interviews (IDIs) and photovoice methods. The study population were YFSWs aged 18–24 years and SRH program implementers in Mwanza city. Mwanza city, a peri-urban area in Northwestern Tanzania and is located on the Southern shores of Lake Victoria. It is the second largest city in Tanzania after Dar es Salaam and as per the last census (2012) has a population size of 722,592 (Mwanza city council, 2012). As of 2016, the estimated GDP/capita in Mwanza region was USD 13,748 (9.7%), compared to USD 24,129 (17%) in Dar es Salaam (National Bureau of Statistics, 2017). The city is a major business and commercial center for trade in regions around Lake Victoria and neighboring countries of Kenya, Uganda, Burundi and Rwanda (Overseas Development Institute, 2016). Mwanza city is one of the fastest growing cities in East Africa (15).

### In-depth interviews

A total of 38 IDIs (30 YFSWs, 8 SRH service providers) were conducted in 2022. The interviews focused on the sexual histories of YFSWs and their lived experiences of seeking HIV and SRH services. IDIs with service providers explored their experiences providing SRH services to YFSWs. All the IDIs were conducted in a private space selected by the participants and were audio recorded with their permission.

### Recruitment of IDI participants

Participants were all recruited from Mwanza city in the densely populated business areas of three suburbs. Since sex work is illegal in Tanzania, we first consulted with sex worker organizations in Mwanza city. We then accessed sex worker networks and informed them about the study. Through collaboration with sex worker networks and two NGOs in the Mwanza city, we mapped out some popular venues where sex workers frequented. Mapping of the sites was done in consultation with local service organizations. Using the time allocation approach (TLA) (16), potential participants were identified and selected during work times (5.00 p.m. to 11.00 p.m.) at venues such as bars, and popular clubs, where YFSWs visited. Four researchers (2 male, 2 females) spent time at the venues informally getting to know YFSWs and their clients and later approached some potential study participants and informed them about the study and set up appointments for conducting IDIs for those who were willing. While at the bars, we also contacted taxi drivers, bar and club owners who sometimes worked as pimps for YFSWs. After initial contact with the first group of YFSWs (4 in each of the three suburbs) accessed through the TLA, we used snowball sampling to gain access to more networks of YFSWs (approximately 12 friendship/peer networks) for further interviews. We also sought their permission to be followed up later to participate in the photovoice component of the study. The eligibility criteria for inclusion in the study for the young women who sell sex was aged 18–24 years, self-identified as selling sex either full time or part time, willing to have an interview; and able to provide informed consent.

Four program leads/coordinators were recruited from two NGOs and two public health facilities that provided SRH services to young people and sex workers in the study site. Three IDIs were conducted with the health providers and two with the lead staff from the NGOs after seeking informed consent.

### Photovoice

Photovoice is a visual research method that involves giving participants cameras to help them to document, reflect upon and communicate issues of concern, while stimulating social change (17–19). This method can be a potentially empowering method because it allows participants to be more in control of the processes of the research such as data collection, analysis and dissemination (17, 19). Through its use of photographic representations that are provided by the study participants, it allows researchers to gain a more in-depth and contextual account of how particular social representations shape up (19). Photovoice is particularly important because of the emotive nature of the photographs which potentially are powerful in exposing and exploring deepening levels of economic and social vulnerability of selling sex. The method is rooted on the assumption that people themselves can best identify and represent their own realities, explore life narratives of selling sex while navigating their lives in specific communities, including their many and diverse relationships with others and the way in which they understand health (and health risks), including seeking and accessing SRH services.

Participants in photovoice were each informed regarding how photovoice works, how to take photos and the safety of the camera and later given a camera and asked to take photos in their context exploring all aspects of their lives such as economics, health and social dynamics. The participants stayed with the camera for approximately 10 days. Photovoice topics were informed by the analysis from IDIs and were guided by two central questions: (1) What is it like to be a young female sex worker living in your community? and (2) In what ways has your experience being a sex worker affected your life and your health and SRH seeking behaviors?

### Photovoice recruitment and training

Through purposive sampling a total of 10 young women who sell sex in the designated age range from ongoing SRH programs (and who had participated in IDIs) were then requested to participate in the photovoice study. Their selection was based on availability and willingness to participate in an additional research activity. After giving their consent to participate in the photovoice study, YFSWs were invited to attend a workshop at the community Center, a site that they had selected as suitable for this. The objectives of the study and procedures for photovoice were explained to all participants. Participants were also trained in using the camera and the ethics of taking photos before being given permission to take photos of interest to them and that represent their situation. Participants were asked to take photos over a 4 week period. Two researchers followed up with participants regularly over the 4 weeks period to discuss experiences on taking photos in the field, while noting challenges, providing guidance and documenting emerging narratives based on participant’s experiences. Once fieldwork was completed, participants were invited to the same venue to participate in four group discussions based on selected photos. The group discussions took approximately 90 min and participants were given refreshments and compensation of $5 for their time. We limited each group to five participants to effectively facilitate the discussions. Two trained researchers moderated each group discussion which lasted 2 hrs. Each group was moderated by two experienced researchers.

## Data analysis

*IDI and photovoice group discussion* tapes were transcribed verbatim, and transcripts checked for accuracy prior to translation. The initial deductive analysis was guided by the photo-topics and evaluation group discussion questions resulting from photovoice.

Data was transcribed by field workers and later translated in English. Transcripts were uploaded into NVIVO qualitative software in preparation for the data coding process. The research team first read and re-read each transcript and a master coding scheme was developed. Coding began with an open coding approach using participants’ language and combining emerging concepts with preconceived theoretical constructs. Constant comparative techniques were used to analyze the data (20). Based on data from an initial 10 interviews (8 YFSWs, 2 with program implementers) and reading of the two photovoice group discussions, an initial set of “open codes” were inductively developed. At the second stage, axial coding was used to group the open codes into more abstract conceptual categories. Axial codes were compared to generate an integrative analytic scheme. After several iterations of analysis, the researchers settled on a final set of four themes: (1) context of sex work for YFSWs; (2) nature of SRH services available to YFSWs; (3) access points of SRHs and (4) an enabling environment for accessing SRH services.

## Findings

### Participant socio-demographics

The YFSWs interviewed were aged between 18 and 24 years with a median age of 21 years. Their daily earnings from sex work were very low, with most reporting earning between $0.50 (Tshs 1,000) and $5 (Tshs 10,000) per sexual encounter. Most of the YFSWs struggled to meet their basic needs with approximately 83% (25/30) reporting not having enough money in the last 1 month. While majority of YFSWs reported that they were 100% sex workers, 5/30 (16%) reported engaging in sex work part time. Majority (25/30) of the YFSWs reported having been in sex work for more than 1 year. The most frequent clients for YFSWs were truck drivers (75%) and policemen with these partner categories overlapping with each other as well as with other groups such as businessmen. The YFSWs in the study were either employed in the bars and sold alcohol and food, while others engaged in petty trade.

The analysis highlighted several themes concerning YFSWs’ experiences of selling sex and their interaction with the SRH services in their communities. Our analysis covers the context of sex work, SRH services available in their communities, barriers and enabling environment for uptake of SRH services.

### “Selling sex when it is illegal”–context of sex work for young female sex workers

Participants reported that sex work was illegal in Tanzania and engaging in it is considered immoral. Hence, all the YFSWs operated discreetly in dark places at night, around alcohol selling venues where they sometimes had temporary employment and had access to clients who came to buy alcohol from such venues. A few operated with the help of pimps at alcohol selling venues. The street based YFSWs reported that they were highly stigmatized and in constant fear of police arrest if found on the streets. Those employed in bars, camouflaged as bar workers while using the venues as hideouts from police. Reflecting on photos taken through photovoice to show context of their work, YFSWs reported:

*During night most of us use those wrecked vehicles to have sex with clients but importantly as our hiding place from the police… When you are inside, it is difficult for the police to know that you are in there… So, while the other girls who are outside are arrested, you are safe… By the time the police leave the place you are through with your business [sex] and get outside [Photovoice group discussion 4]*

Most of the YFSWs reported that selling sex was the main way through which they earned income. The few, who reported selling sex as a part time activity were also involved in petty trade such as food vendors and working in beauty salons. Such women used their involvement in other businesses as a cover up for their engagement in a trade considered “illegal.” Since the other ‘legal’ economic activities could not sufficiently sustain the economic needs of most YFSWs, they engaged in sex work to cater for their basic needs such as food and shelter.

*I do this business of selling sex out of need… We do this business of selling sex, to get money to buy food… But also, you must pay rent… Your children all depend on you and the money is not enough. [Photovoice group discussion 2]*

Almost all YFSWs worked in an environment of shame and disregard for the profession of sex work. YFSWs reported that although they engaged in sex work it is something they too perceived negatively but was a necessity for sustaining them. They talked about constantly being embarrassed for engaging in sex work:

*I feel bad for being in this business… It is embarrassing, and I honestly feel bad when people in the community see you and point fingers at you… Now you find that some have husbands who provide for them. But for us, we are forced to do this for our children and families… We feel embarrassed about what our children see and hear, I mean we feel bad. [Photovoice group discussion 2]*

The illegality of selling sex meant that police brutality and sexual violence was commonplace for YFSWs. Almost all (29/30) YFSWs reported that they had been victims of sexual violence including rape, physical violence from intimate partners, clients as well as from total strangers. Despite reported high levels of violence, YFSWs rarely acted due to fear of arrest as police were also key perpetrators. Many YFSWs talked about being harassed by police and when they were arrested, were forced to have oral and vaginal sex with the police officers.

*When the police arrest you and lock you up, they now start looking for the one who is beautiful among you. He tells you to suck his penis so that he can free you. Now that is a challenge… Or he makes love with you and doesn’t pay you anything. They also snatch your money and your phones…. Like during the month of December we are usually arrested and taken there [police station] [Photovoice group discussion 1]*

Further reflecting on working in an environment of violence and police brutality, YFSWs talked about not having anywhere to report the daily mistreatments they received while on the streets because when they tried reporting the police accused them of engaging in illegal business and risked further abuse.

*It is not just an environment of being mistreated by customers, but also police brutality. You think that a police station is supposed to be the safest place to run to… but no, it is very bad. So, you wonder, now who will you report to?… If you have money, the [police] take it all and leave you with nothing. [Photovoice group discussion 3]*

Given the precarious nature of sex work, YFSWs described some guest houses in their communities as common areas that were considered as relatively safe schooling grounds for younger women starting sex work. These houses were seen as steppingstones into sex work. Using a picture taken during photovoice, they described this as:

*This picture is of a certain guest house which is very famous in our suburb. A big percentage of girls who come from the villages normally stopover in this guesthouse… I mean those who want to do commercial sex business normally make a stop in this guest house…. That is why I have taken this photograph and made it my first. When I came from the village it was my first stopover and I started my business from there [Photovoice group discussion 1]*

The illegality of sex work affected how and whether YFSWs negotiated for condoms use with clients. While majority of YFSWs (24/30) reported that they understood the importance of sex workers using condoms, only a few (8/30) reported using condoms consistently. YFSWs mentioned that when they first meet a client, they were pre-occupied with negotiating for their pay than negotiating for the use of condoms. They left the responsibility of deciding on whether to use a condom or not to the client.

## Nature of sexual and reproductive health services available in the community

Majority of the YFSWs had good understanding of the SRH vulnerabilities faced by women who sell sex. They talked about client violence, rape, risks for infection with sexually transmitted infections such as HIV; risks for unplanned pregnancy and risks for being killed. Many YFSW were more concerned about wanting to prevent HIV and unpanned pregnancy than the other risks. The YFWS were single but with children and did not desire another pregnancy due to the economic challenges they faced in fending for their children. The economic stressors pushed them into further vulnerability.

YFSW mentioned the commonest SRH services they needed as being HIV testing and treatment, HIV prevention, family planning, STI services and medication for drug addiction. Although some of these services were available in their communities, none were specific for YFSWs. Health providers reported that YFSWs were expected to access these services like other community members. YFSWs reported that they accessed SRH services from: local pharmacies, government public health facilities, peer sex workers, and through NGO community outreach activities. Two organizations worked in the study communities offering HIV counseling and emotional support for YFSW but in case of any major services such as HIV treatment, they were expected to visit the district hospital. One’s HIV status determined frequency of contact with health facilities with HIV positive YFSWs reporting more contact with services than those who were HIV negative.

### HIV/AIDS services

YFSWs reported that during their routine HIV treatment services, they also received information on pregnancy prevention and the need to be extra cautious with their health:

*Due to my present situation of being HIV psositive, they instructed me what to do. They tested my CD4 and advised me to wait for three or four years before getting pregnant… They usually advise us to use protection and give us contraception. [IDI, 24 years old, HIV+, single, with children]*

Most of the YFSWs reported that they had never used contraception for varied reasons ranging from provider related to client drawbacks. Examples of provider related barriers were their recommendation that they use a family planning method that was not their choice and the unfriendly attitude of some health providers. Client related factors involved: social norms that encouraged them to have children; fear of family planning causing infertility among young women who have never given birth; stigma because of engagement in sex work; provider beliefs that family planning was not suitable for young women without children and provider preference for certain methods that were unpopular among YFSWs. The providers’ views were in line with those of YFSWs, especially those who reported not using family planning. They believed that contraception could cause infertility and thus curtailing their future aspirations to bear children.

One woman described her experience seeking family planning in the following:

*I have never gone back there because the first time I went there to ask for family planning, they told me that I can’t get that service if I have not given birth [IDI, 19 years, single]*

### Family planning services

Health providers mentioned that although the health facilities where they worked had the option of long-acting methods, they reserved those for older married women with children. Health providers preferred to prescribe short-term methods for young women including those involved in sex work. One provider from a government health facility talked about the societal expectation for young women to bear children and thus justifying her reason for discouraging young women from using family planning in the following:

*The long-term methods like loops [IUD] stop fertility… We can’t advice the young ones to stop fertility because you also consider the age of that girl. She might get a man who will marry her. She will have her life in future. So, we advise her against those long- acting ones and instead give her the short-term methods. [IDI, Health provider]*

The few YFSWs who had been successful in accessing family planning mentioned that they had encountered challenges getting the services. They reported that they sometimes needed to visit the health facility more than twice to obtain the services. Out of desperation, some said they were forced to bribe the health providers so that they could receive the services quickly. A young woman who sought services from a public facility recounted her experience in the following:

*…You must get on the queue… I went on the first day and didn’t get it. I had to go again for the second time and that is when I got the injection… There are no costs for the services but if you want to maneuver through the long queue then you have to bribe the nurse… You can even get there at 8.00 in the morning and you are served at 4.00 in the evening… therefore you find a nurse whom you can give two thousand shillings ($1), and that’s when she will listen to you and rush you to get an injection… I mean she immediately takes you to that service. [IDI, 22 years, single]*

Due to challenges at the public health facilities, some YFSWs reported that they preferred long-acting contraception to avoid frequenting the facilities. They also talked about some of the long-term ones being easy to hide from their partners. A few reported that they resorted to tactics such as lying to the health provider about their marital status and the number of children so as to get their preferred long-term family planning method.

*They asked me how many children I had, I lied because if you have one child then you’re not allowed to put these contraceptive sticks [implants] of five years… I told them I had two, it’s when they gave me that one for five years [IDI YFSW, 24 years, single, without children]*

YFSWs switching between family planning methods was common. Many gave the reason for switching between methods as a strategy of finding one that worked conveniently for their sex work profession. Due to the pressure to have income every day, many talked about preferring a family planning method that stopped menstruation so that they could be able to meet clients daily. A YFSW with children reported:

*I started off with the injection, then I went to pills and now I am on sticks [implant]… …. I don’t want to have a baby now because I will mistreat the baby…My life is not good. My work involves having sex with ten men daily, without rest.… I am on family planning but still menstruate and that is the only hinderance to having sex…I would prefer one that stops that bleeding. [IDI, YFSW, 24-year-old, separated, with children]*

### From facilities to communities: YFSWs accessing SRH services in the communities

SRH services were sometimes also available to YFSWs through community outreach activities mostly conducted by non-governmental organizations. The community outreach services were available to all community members including sex worker.

*When health workers come to test us here in the community, they counsel us and give us condoms. They start by testing for HIV and telling you if you are safe [HIV negative] and then they give you a condom. [IDI, YFSW, 2years old]*

Outreach services mainly related to HIV testing were available in communities on National and international public health holidays such as World AIDS days and a few select days.

*They come to that center [community outreach] to test us test for the HIV/AIDS. I remember they came last year. Many of my friends tested. But I know myself and my secret. Because most of the people went for testing, I did not go because I did not want my secret to be revealed [about HIV positive status, [IDI, YFSW, 19 years]*

Condoms were the most common SRH service that was easily accessed outside the healthcare system. Access to condoms was also integrated with that of other SRH services. For example, participants mentioned that they were offered condoms when they visited health facilities for HIV services such as testing, treatment and family planning services.

*You get the condoms at the clinic when you go for the family planning injections [IDI, YFSW, 22 years, with children]*

A second participant reported how she accessed condoms when she went for her antiretroviral therapy:

*Like at the place where we go to get HIV medicine [ART], when you ask them for the condoms, they just give it to you. [IDI, YFSW, 20 years]*

The few YFSWs who reported using condoms mentioned that they preferred to obtain them from retail shops and pharmacies rather than from health facilities. While condoms were provided for free in public health facilities, they cost Tshs 500–100 ($0.25–$0.5) per packet of 3 condoms in the community shops and local pharmacies. Most YFSWs mentioned that they were willing to pay for these services at pharmacies and retail shops than getting them for free from public health facilities. A YFSW who preferred getting condoms from the village retail shops reported:

*I usually pass by the shop and buy some condoms worth five hundred ($0.25) and take them with me to work. [IDI, YFSW, 23 years]*

YFWs talked about prices for condoms varying by quality. They believed that softer condoms were better quality and cost more than the free types provided in public health facilities that were often dry. They gave examples of brands that were available in their communities as Salama, Bull, Kipindi, Fiesta, La Vida and others. YFSWs however acknowledged that despite retail shops and pharmacies having variety and selling good quality condoms, they were more expensive compared to public health facilities.

*We have those that cost five thousand [$2] and those that cost ten thousand [$5] up to more than ten thousand [more than $5]. The one that costs five thousand [$2] is normally dry and the one that costs ten thousand [$5] is somehow slippery but the one that cost fifteen thousand [$7] is very soft. [IDI, YFSW, 22 years]*

Other community places where YFSWs accessed condoms were retail shops, pharmacies, and guest houses/lodges. YFSWs mentioned that having prior good relationships with shop owners facilitated access to condoms. They talked about sometimes getting condoms on credit or at a discounted price from shops with friendly owners and where they had become frequent customers.

*I normally buy condoms from a certain retail shop. The owner is now used to me and considers me his customer. I have known the owner of that shop for a long time, and he now sells me a whole packet at a discounted price. [IDI, YFSW, 22 years]*

A few YFSWs reported that they accessed condoms from the guest house facilities. Some guest houses and lodges provided condoms for free on condition that the YFSW used their facilities with their clients.

*So long as you take a room there, they provide you with condoms. They just give you for free [IDI, YFSW, 18 years]*

Despite YFSWs preference for accessing condoms from the community, they reported that this was limited to daytime service as the shops and pharmacies were closed at night-time and yet that was the moment when they needed condoms most.

*You find that the condoms that you have get finished at eleven in the night… Now what pharmacy is not open at that time? [IDI YFSW, 23 years old]*

NGOs were also important access points for community distribution of SRH targeted at YFSWs. YFSWs mentioned the two NGOs that provided services to them as ICAP, an American led NGO and Cheka Sana a local NGO. Majority of participants reported receiving HIV education, HIV testing, lubricants and condoms freely from ICAP:

*We normally get those services from ICAP whenever they come for a SRH seminar here [community bar]. Sometimes they come behind there or here at X. They sometimes test us; they educate us and give us condoms together with that KY oil [lubricants] [IDI, YFSW, 24 years]*

### Stigma as a barrier to accessing SRH services

Despite SRH services being available in some health facilities and community outreach venues, stigma was a huge hinderance for YFSWs accessing these services. The majority feared going to busy SRH facilities unless they had a major health issue that required them to visit such places. YFSWs reported variations in SRH services provided at the health facilities in their communities and hence preferred some facilities over others. While public health facilities were expected to provide services for free, others charged a fee for acquisition of files for keeping personal records. Women seemed to prefer facilities that provided services for free.

*I prefer place X [maternal and child health clinic] because you get treatment for free. They also have pharmacy on site… Everything is free… But now when you go at dispensary Y [private clinic], every little thing you pay money… you pay for the file… you also pay for the medication. But at the public facility, all the services are free. [IDI, YFSW, aged 19 years]*

Health providers reported that most young women including those engaged in sex work sometimes experienced self-stigma when accessing SRH services. Many feared approaching providers to seek these services because of what people would think about them if they were seen in the HIV department at the health facility.

*These girls experience shame… They always think “what will people say about me? They will say that I am infected with HIV.” People will talk about me and say I have AIDS” [IDI, NGO staff]*

While at the health facility, the facility environment was also key in determining whether YFSWS were motivated to use the services or not. Stigma wasn’t just experienced from the provider, but also within the environment around the facility. YFSWs talked about unfriendly non-medical staff such as security guards who questioned them when they entered the health facility compound. One woman reported:

*The guards also insulted us. When they see someone sitting down, they tell you all sorts of rude things… there are lots of discouraging things there [IDI, YFSW, 18 years]*

### Peer influencers creating demand for SRH services for young female sex workers

Peers were important in influencing other YFSWs’ choice of SRH services and where and how to access the services. YFSWs mentioned that they shared their supply of condoms with other young women and advised them on what services to use and where to find it.

*My friend and I discussed and decided that we should go to X (Maternal and Child health clinic). We advised each other to use family planning or condoms so that we do not become pregnant unexpectedly since we do not have permanent boyfriends… Since we are with men to meet our economic needs, we decided together on ways to prevent pregnancy. [IDI, YFSW23 years]*

Peers were also an important access point for provision of SRH services, especially condoms. This was particularly for the peers who worked in health services:

*A fellow sex worker who also happens to be my friend, works at the referral hospital. She usually brings me boxes of condoms. [IDI, YFSW, 24 years]*

NGO staff reported that they had several ways of supporting girls and examples of this were through peer support clubs. One NGO for example provided psychosocial support to young women infected with HIV by facilitating meeting through peer support groups. Young women living with HIV including YFSWs were encouraged to attend these sessions for peer support. One implementer from one of these organizations reported:

*Our work has built the ability of girls to believe in themselves… that even though I am HIV infected, life must continue… So that has motivated us to continue helping them… They have joined different clubs where they learn and are supported by their peers. [IDI, NGO coordinator]*

### Building an enabling environment to motivate YFSW to access SRHs

Both health providers and YFSWs mentioned that respect and trust must be built between the health providers and YFSWs. Health providers admitted that it was important for them to be approachable and trusted by young people, especially those who sell sex if they had to support them in consistently seeking and using SRH services. Health providers mentioned that many of their peers lacked the necessary skills of engaging YFSWs and said that they needed to be empowered with the relevant skills. Health providers could not differentiate young people who engaged in sex work from those who did not, when they visited the health facility. One health provider talked about her experience of getting to know her client, a YFSW in the following:

*It is difficult to know if she engages in that business during her first visit. So, she just gets the services like any other person…. Sometimes you need extra skills to probe until you know they engage in that practice… they don’t want to be recognized that they do that business …In the beginning she will tell you that I do small scale businesses, I sell this and that… but with continuous education and engagement with her, she can later disclose that she sells sex… Building that trust is important. [IDI, health provider]*

Most health providers recognized the challenges faced by young women especially those who engage in sex work and described the need to show young people respect, the need to be patient centered when serving YFSW and to avoid being judgmental when they visited the health facilities. One SRH provider reported:

*You know, there are people that don’t tolerate insults… Like those sex workers, sometimes they are rude, but you must be patient with them and take them as they are… When you talk to her you should not judge or stigmatize her. [IDI, health provider]*

Health providers further added that YFSWs are a group that would not like to be seen hanging around health facilities seeking SRH services for fear of stigma linked to HIV infection. One provider said that it was important to serve YFSWs workers quickly so that they could leave the health facilities.

*Due to the nature of their work, they want to be served quickly and so that they can leave that place [health facility]… So if you make her wait for maybe a long time, she won’t like it… she will leave without the service or not return… I would say their [YFSWs] services are supposed to be quick but very friendly… just like how you handle a child, then you’re supposed to handle them the same way. [IDI, Health provider]*

Views of YFSWs aligned with those of health providers with all reporting that they desired health providers who are friendly and not judgmental. One woman expressed her desire to receive SRH services from facilities with friendly and polite providers in the following:

*Those who give the services, like the nurses should be friendly and polite… But not those who shame you when you get there [health facility] …those are not appealing to anyone. [IDI, Health provider]*

Localization of SRH to the venues where YFSWs work is key. When asked how best to increase access to SRH services for YFSWs, health providers reported that there was need to target services to places where they meet clients. Health providers also mentioned a need for specialized services for young women who self sex. Giving an example of an NGO that was good at providing services in the places where YFSWs station themselves for clients, one provider reported:

*There is a need for people and organizations that follow them. For example, ICAP, follows them at the places where they work …So that is one of the ways…They now start to appreciate that people care about them. [IDI, health provider]*

## Discussion

The lives of YFSWs are riddled with complexity, with social locations and structural forces interacting to shape their experiences. We set out to understand the context in which YFSWs operate and how they access SRH services in Tanzania. Given that sex work is illegal in Tanzania, YFSWs operated discretely. The YFSWs in our study operated on streets/roadsides, around bars, alcohol selling venues, and around lodgings known to have clients. They were hard to reach with stationed SRH services as most were mobile and kept changing venues based on client availability and police presence. Studies have noted the difficulty in reaching informal sex workers especially those who work on streets (21–23) with SRH services. Most struggled to access HIV services leading to non-adherence to HIV treatment for those who were living with HIV. Our findings are similar to what was observed elsewhere indicating poor engagement to HIV care and treatment among female sex workers (9, 12). In order to ensure sustained reach and use of SRH service among YFSWs in Tanzania, there is a need to employ flexible approaches such as mobile clinics, clinics being open late in the evening, use of peer to provide services where possible.

Young female sex workers operated under difficult economic circumstances with many reporting they engaged in this business to earn a living and to support their children. The precarious lifestyle experienced by these women, meant that many did not prioritize negotiation of condom use with clients but instead focused on getting compensated for their services. The negotiation around condom use was further complicated with the illegality of the sex work that made it difficult for young women to use decent and safe guest houses with clients for fear of police arrest. Limited economic options were major drivers for young women entering into sex work and determined whether they had access to HIV prevention services and contraception. It is important that interventions focus on addressing the structural drivers (24) that push young women into poverty and further into selling sex as a means of survival. Very few interventions (11), have specifically targeted young women who sex in sub-Saharan Africa. This age group needs to be prioritized in economic strengthening and SRH programming to reduce HIV risk.

Stigma around sex work is a major issue in Tanzania (25, 26) and in many sub-Saharan Africa countries (6). Our findings point to YFSWs experiencing violence, stigma, discrimination within and around the health facilities. Such experiences were related to their young age and the expectations for seeking SRH while not married. Stigma was also experienced in relation to seeking HIV services, which was a clear pointer that they were possibly infected with HIV. Although evidence exists on the role of stigma and discrimination as a barrier oyoung people seeking SRH services (6, 11, 24, 26, 27, 28) there is a dearth of literature on YFSWs. The experience of stigma among young women was further compounded by the fact they were engaged in sex work. This calls for specific services for this age group and community interventions to minimize stigma and discrimination toward young women.

It is apparent from our findings that YFSWs must be empowered to take control of their sexuality and the risks they face. YFSWs in our study sometimes desired to use condoms but were limited by the quality of condoms that were available for free from their communities (e.g., little lubrication) as well as the venues that they were expected to access them from. Most preferred to get their supply of condoms from local retail shops and private pharmacies, which seemed to stock better quality brands and had good customer care services than in public health facilities. This implies a need to increase options and venues where YFSWs could access condoms. There is also a need to address the issues around perceptions of condom quality so that the YSFWs can have a desire to use the free condoms provided in the public health facilities if they decide to. Studies have shown that condoms use interventions among sex workers reduce their HIV risk (11, 29) and hence promotion of condom use is a worthwhile intervention among YFSWs.

Our findings show that there were no specific SRH services for sex workers in many health facilities. YFSWS experienced long waiting hours to access services and were sometimes forced to bribe through the system to get services. As noted in other studies (6, 28, 30), we also found that long waiting times for HIV treatment increased their stigma further discouraging YFSWs from using HIV services. In order to expand access to SRH services, there have been recommendations for differentiated services for key populations including YSFWs in (31). Since services targeting YFSWs must differ in intensity from services that target general population at lower risk, differentiated care could enhance YFSWs access to SRH services.

YFSWs demand for SRH services was not uniform but depended on many factors such as age and economic demands. While the older ones with children expressed a need for family planning services, the younger ones did not seem to have a similar priority. YFSWS were also not able to get their preferred SRH service. For example, while many preferred long-acting injectable contraception compared to short-term ones, the providers were reluctant to recommend such services for them due to the belief that they were still young and did not have children and that certain contraception could cause infertility. Given this reasoning many YFSWS were unable to get the contraception of their choice and instead were forced to use what the provider chose for them. This finding is similar to what has been noted in other studies on adolescent girls and young women accessing services in sub-Saharan Africa (32). As observed in our findings, the fears about contraception delaying births and causing infertility are not just limited to YFSW but have also been reported in other studies among young people in Tanzania (33). Such beliefs need to be addressed by appropriate interventions targeting health providers so that they can support YFSW to easily access their preferred contraception (14). In line with the WHO essential package of SRH services (34), there is also a need for other SRH services like those on prevention of gender-based violence, sexuality education and psychosocial services for adolescents as they transition into adulthood.

Trust is an important aspect for seeking SRH services (31) among key populations including sex workers. YFSWs preferred to get advice on SRH services from peers that they trusted than from expert health providers that were not friendly. Peers were in a similar situation to them and were considered not judgmental and more empathetic than many SRH providers. They also opted to purchase condoms from retail shops and local pharmacies that they trusted instead of getting such services for free through public health facilities. There have been calls to involve young people in the co-design of their interventions and such should be extended to YFSWS whereby programs involve them in co-design and the actual implementation. There has been some progress toward the general sex work population (24, 25) but not much consultation has occurred to target the specific group of YFSWs.

Since we collected data on a sensitive subject within a group that is highly stigmatized in Tanzania and operating in a profession that is illegal, it is likely that YFSWs were not able to express their views on some of the sensitive questions around their sexual experiences. This was however minimized by using researchers who were experienced in collecting data with key populations including sex workers. The utilization of photovoice method for data collection was also very empowering (17, 19) to YFSWs as it allowed them to take control of the data collection process and to present data that they were comfortable to share with the researchers through the photos they took. We would highly recommend the use of photovoice in the collection of data with YFSWs. This method allows for more individual level reflection and collection of data that speaks to the situation of sex work especially in settings where the practice is highly stigmatized and where intimate partner violence is prevalent.

### Credibility of findings

We attempted to ensure the credibility of our findings via several approaches. First, we used prolonged and repeated engagement to build rapport with participants as well as probing their responses to encourage interviewees to give examples to support their statements. Second, we triangulated qualitative methods allowing for complementarity and all rounded understanding of the research questions. While IDIs allowed for the individual level reflection on personal experiences, photovoice provided detailed information on context of sex work within the settings where the YFSWS operated in. Third, we engaged three researchers to ensure reliability in coding and theme development. Fourth, we validated our initial findings by discussing the findings with participants during group discussions for clarification and to check on whether they agreed with our interpretation of the findings.

### Reflexivity

Reflecting on our potential role on the research process and findings, it is worth noting that all the authors had worked in SRH over the years and two lived in the research communities. It is possible that we may unintentionally have biased the findings by how we interpreted them given our prior understanding of the health care system. However, the senior author on the study did not have prior engagement in the study setting.

## Conclusions and policy implications

YFSWS operate under very stressful conditions and have limited access SRH services in Tanzania. Peers of YFSWs played a key role in accessing and using SRH services and could be utilized to promote access to services among YFSWs network.

Given the numerous challenges faced by YFSWs in accessing SRH services, interventions targeted at this group should focus on multi-level barriers to engagement with services. These could be addressed at different levels as follows: (a) health facility level with differentiated type of SRH care/services that are friendly and trusted by YFSWS; (b) community level to address stigma and social norms that promote gender inequalities and that stigmatize YFSWs; (c) individual level by providing economic livelihood options and SRH knowledge to YFSWs; and finally, (d) at policy level by addressing the policies that criminalize sex work in Tanzania and building an enabling environment to motivate YFSWs to access SRHs.

## Data Availability

The raw data supporting the conclusions of this article will be made available by the authors, without undue reservation.
